# Detection of Activation Sequences in Spiking-Bursting Neurons by means of the Recognition of Intraburst Neural Signatures

**DOI:** 10.1038/s41598-018-34757-1

**Published:** 2018-11-13

**Authors:** José Luis Carrillo-Medina, Roberto Latorre

**Affiliations:** 1 Departamento de Eléctrica y Electrónica, Universidad de las Fuerzas Armadas - ESPE, Sangolquí, Ecuador; 2 Grupo de Neurocomputación Biológica, Dpto. Ingeniería Informática, Universidad Autónoma de Madrid, 28049 Madrid, Spain

## Abstract

Bursting activity is present in many cells of different nervous systems playing important roles in neural information processing. Multiple assemblies of bursting neurons act cooperatively to produce coordinated spatio-temporal patterns of sequential activity. A major goal in neuroscience is unveiling the mechanisms underlying neural information processing based on this sequential dynamics. Experimental findings have revealed the presence of precise cell-type-specific intraburst firing patterns in the activity of some bursting neurons. This characteristic *neural signature* coexists with the information encoded in other aspects of the spiking-bursting signals, and its functional meaning is still unknown. We investigate the ability of a neuron conductance-based model to detect specific presynaptic activation sequences taking advantage of intraburst fingerprints identifying the source of the signals building up a sequential pattern of activity. Our simulations point out that a reader neuron could use this information to contextualize incoming signals and accordingly compute a characteristic response by relying on precise phase relationships among the activity of different emitters. This would provide individual neurons enhanced capabilities to control and negotiate sequential dynamics. In this regard, we discuss the possible implications of the proposed contextualization mechanism for neural information processing.

## Introduction

From a functional point of view, action potentials or spikes are informational events that allow individual neurons to compute and communicate by transforming synaptic input into output spike patterns. A common feature of the temporal organization in the firing pattern of many neurons consists of grouping individual spikes into *bursts* separated by quiescent periods in the so-called *spiking*-*bursting* activity^1^. The role of burst firing has been discussed in the context of many different neural systems. Bursts of spikes have been traditionally considered as unitary events that are treated as a whole by the reader of the neural signal. From this perspective, the importance of the slow depolarizing wave in neural bursting behavior and the role it plays in the communication between bursting cells is well-known (e.g., see refs^2–7^). Nevertheless, spiking-bursting activity involves the presence of at least two different time scales that can serve to encode distinct informational aspects: one related to the slow depolarizing bursting period and another related to the fast intraburst spiking timescale. Only in recent years the role of fast dynamics in bursting neurons is receiving some attention. For instance, experimental and modeling studies have addressed the encoding of different stimuli by means of specific intraburst spike patterns (IBSPs)^8,9^; the effect on the muscle response of certain intraburst properties such as the interspike frequency or the number of spikes per burst^10–13^; the existence of channel-specific information discrimination mechanisms at the single-cell level depending on the timings within a spike train^14,15^; or the selective response of a postsynaptic neuron to specific interspike frequencies^16,17^. Of particular interest in this context is the observation of robust cell-specific intraburst firing patterns in both invertebrates and vertebrates neurons^18–21^. These characteristic IBSPs can be considered a *neural signature* that allows us to identify the signal source. Some intraburst signatures are robust and reproducible even across different species^22^. The observation of these fingerprints in widely different neural systems and their conservation in evolution raise several intriguing questions related to the existence of mechanisms to identify the origin of a neural signal and to the information processing based on this identification. Furthermore, the generation of a neural signature in living cells coexists with the encoding of information in other informational aspects of the bursting signal, e.g.,the slow depolarizing wave. This hints at the use of a multiplexed encoding strategy where the neuron identity could be transmitted together with a content message^23,24^. The use of multiplexed codes in the same signal has been discussed in different sensory and motor networks^25–30^. Multicoding strategies for information propagation can greatly enhance the computational capacity of neural systems, as they allow transmitting and processing multiple information simultaneously^31–34^. The multiple simultaneous codes can be processed one-by-one or simultaneously to perform different tasks, and not all the readers of the signal have to be interested in the same informational aspects^23,24,35^.

In this paper, we are interested in the possible functional meaning of intraburst neural signatures. Although their role in neural computation is still unclear, previous experimental and modeling results suggest a relevant functional significance for the systems where they are present, in particular for central pattern generators (CPGs)^22,23,36–38^. CPGs are assemblies of neurons that, acting alone or together with other CPG circuits, produce sequential patterns of bursting activity to drive motor function^39–41^. We hypothesize that the ability of neural systems to “sign” their outputs and identify the origin of their inputs would have significant implications for neural sequential dynamics, leading to a more selective and complex information processing. Sequential dynamics usually underlies what is termed as rhythm or spatio-temporal pattern of activity. These are essential for the organization of complex behaviors in invertebrates as well as in vertebrates: from the alternating patterns of activity generated by CPG circuits in activities like breathing, chewing or swimming^42–46^; to the complex sequential dynamics in the brain for perceptive, cognitive and motor processing^47–58^. Unveiling general principles in the generation and coordination of robust sequences of neural activations is therefore a highly relevant topic in neuroscience. From this view, the departing hypothesis of this investigation was that cell-specific IBSPs may not only allow a postsynaptic cell to contextualize incoming messages and selectively react to input from specific emitters, but also to detect and discriminate specific activation sequences among different presynaptic units. Note that this requires a multiplexed encoding for information propagation regarding the “who” (neuron identity) and the “what” (sequential dynamics) of the information. In general, living cells receive many inputs from different sources. In this scenario, if emitters encode a characteristic intraburst signature in their output, readers of these signals would receive multiple of these fingerprint simultaneously (or very close in time) through its synaptic afferents. Some specialized reader could then use incoming signatures to characterize the collective sequential activity and produce a coherent response accordingly. In particular, neural signatures would allow the reader to selectively process specific activation sequences as a function of the neurons participating in the sequence and the phase relationship among their burst firings.

To address our hypothesis, we used a detailed biophysical neuron model with a rich spiking-bursting behavior and study its response to the reception of sequences of signed bursts from different emitters. We consider that this reader cell is able to detect and discriminate specific activation sequences when, independently of the slow wave frequency of the input rhythm, a selective input-output transformation arises depending (i) on the intraburst signatures encoded in incoming signals, i.e., on the participants in the rhythm, and (ii) on their relative firing timings. Our simulations suggest history-dependent information processing capabilities associated to each input channel with fine temporal sensitivity at the subcellular and synaptic level. Information processing in the reader takes place in two simultaneous dimensions. On one hand, intraburst signatures allow the postsynaptic neuron to contextualize the information received through each synaptic afferent as a function of the signal source. On the other hand, the temporal processing of the spike trains arriving through the different input channels allows building complex input-output relations depending on the relative activation timings of different groups of presynaptic cells. The existence of such discrimination mechanisms in living systems would have relevant computational implications for the neuron, since they would permit single cells to selectively react to specific activation sequences beyond simple resonant responses. This is a highly valuable feature for the control of sequential dynamics that would have significant implications for the sequence negotiation, i.e., the process of determining participants and timings in the sequential activity.

## Materials and Methods

### Characterization of neural signatures

Intraburst neural signatures are characteristic IBSPs within the spiking-bursting activity produced by a neuron^18,19^. Their temporal structure can be characterized by the corresponding intraburst interspike intervals (*ISI*_*i*_). Then, to expose the signature encoded in a bursting signal, we built raster plots representing the firing times within the sequence of bursts in the signal. Neural activity in these plots was aligned according to the first spike in the corresponding burst. This graphical representation allowed us to visually compare differences among the signature of different neurons. Additionally, to quantitatively measure how similar two signatures were, we used the following L2 norm^23^:

1 $${d}_{{S}_{1},{S}_{2}}=\sqrt{\frac{1}{{B}_{1}\,{B}_{2}}\,\sum _{i}^{{B}_{1}}\,\sum _{j}^{{B}_{2}}\,\sum _{k}^{N}\,{(IS{I}_{k,i}^{{S}_{1}}-IS{I}_{k,j}^{{S}_{2}})}^{2}}$$

where *B*_1_ and *B*_2_ are the number of bursts in signals *S*_1_ and *S*_2_, respectively; and being *N* the number of ISIs per burst. Note that this measurement required of the burst of both signals to have the same number of spikes.

### Models

All equations of our models were numerically solved with a Runge-Kutta6(5) variable step method with a maximum error of 10^−15^.

#### Neuron Model

Different neuron models, such as the models proposed by Hindmarsh and Rose^59^, Komendantov and Kononenko^60^ or Liu *et al*.^61^, have a demonstrated ability to generate and recognize intraburst neural signatures^23,24,37,62^. To model the individual dynamics of a reader neuron, in this work we used the Komendantov-Kononenko’s proposal. This conductance-based model (proposed for snail CPG cells) includes a slow-wave generating mechanism, a spike generating mechanism, an inward calcium current, an intracellular Ca^2+^ buffer and a [Ca^2+^]_*in*_-inhibited calcium current. Komendantov-Kononenko model neurons exhibit the rich slow-fast dynamics observed in the spiking-bursting activity of several living neuron types, which underlies their ability to produce cooperative coordinated rhythms^63^. The membrane potential equation is:

2 $$-{C}_{m}\frac{dV}{dt}={I}_{Na(TTX)}+{I}_{K(TEA)}+{I}_{K}+{I}_{Na}+{I}_{Na}(V)+{I}_{B}+{I}_{Ca}+{I}_{Ca-Ca}$$

The slow-wave generating mechanism is given by sodium, potassium and chemo-sensitive currents:

3 $${I}_{Na}(V)={g}_{Na}^{\ast }(V)\cdot \frac{1}{1+exp(\,-\,0.2\cdot (V+\mathrm{45))}}\cdot (V-{V}_{Na})$$

4 $${I}_{Na}={g}_{Na}^{\ast }\cdot (V-{V}_{Na})$$

5 $${I}_{K}={g}_{K}^{\ast }\cdot (V-{V}_{K})$$

6 $${I}_{B}={g}_{B}^{\ast }\cdot {m}_{B}\cdot {h}_{B}\cdot (V-{V}_{B})$$

7 $$\frac{d{m}_{B}}{dt}=\frac{1/\mathrm{(1}+exp\mathrm{(0.4}\cdot (V+\mathrm{34)))}-{m}_{B}}{0.05}$$

8 $$\frac{d{h}_{B}}{dt}=\frac{1/\mathrm{(1}+exp(\,-\,0.55\cdot (V+\mathrm{43)))}-{h}_{B}}{1.5}$$

The spike generating mechanism is described by TTX-sensitive sodium and TEA-sensitive potassium Hodgkin-Huxley type currents:

9 $${I}_{Na(TTX)}={g}_{Na(TTX)}^{\ast }\cdot {m}^{3}\cdot h\cdot (V-{V}_{Na})$$

10 $$\frac{dm}{dt}=\frac{1/\mathrm{(1}+exp(\,-\,0.4\cdot (V+\mathrm{31)))}-m}{0.0005}$$

11 $$\frac{dh}{dt}=\frac{1/\mathrm{(1}+exp\mathrm{(0.25}\cdot (V+\mathrm{45)))}-h}{0.01}$$

12 $${I}_{K(TEA)}={g}_{K(TEA)}^{\ast }\cdot {n}^{4}\cdot (V-{V}_{K})$$

13 $$\frac{dn}{dt}=\frac{1/\mathrm{(1}+exp(\,-\,0.18\cdot (V+\mathrm{25)))}-n}{0.015}$$

The inward calcium transient voltage-dependent current is described by:

14 $${I}_{Ca}={g}_{Ca}^{\ast }\cdot {m}_{Ca}^{2}\cdot (V-{V}_{Ca})$$

15 $$\frac{d{m}_{Ca}}{dt}=\frac{1/\mathrm{(1}+exp(\,-\,0.2\cdot V))-{m}_{Ca}}{0.01}$$

And, finally, the calcium stationary [Ca^2+^]_*in*_-inhibited current is given by:

16 $${I}_{Ca-Ca}={g}_{Ca-Ca}^{\ast }\cdot \tfrac{1}{1+exp(-0.06\cdot (V+\mathrm{45))}}\cdot \tfrac{1}{1+exp({k}_{\beta }\cdot ([Ca]-\beta ))}\cdot (V-{V}_{Ca})$$

17 $$\frac{d[Ca]}{dt}=\rho \cdot (\frac{-{I}_{Ca}}{2F\upsilon }-{k}_{s}\cdot [Ca])$$

where $\upsilon =4\pi {R}^{3}/3$ is the volume of the cell; [*Ca*] is the intracellular Ca^2+^ concentration (mM), *F* is Faraday number (*F* = 96,485 C mol^−1^), *k*_*s*_ is the intracellular calcium-uptake rate constant and *ρ* is the endogenous calcium buffer capacity.

**Figure 1:**
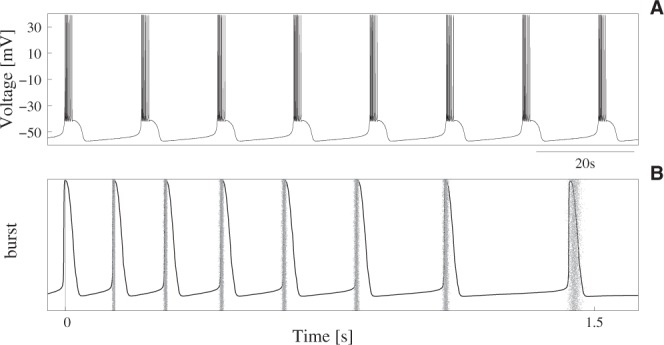
Neuron isolated dynamics. (**A**) Regular bursting activity of a single Komendantov-Kononenko model neuron with the parameters specified in Table 1. With these parameters, and in the absence of synaptic input, the reader neuron produced a highly precise sequence of decelerating 8-spike bursts at a slow-wave frequency equal to 0.13 ± 0.0007 Hz. Initial conditions are: *V*_0_ = −55 mV and [*Ca*] = 0 mM. (**B**) Raster plot characterizing the IBSP of the neuron of panel A in a time series containing 5000 consecutive bursts. Action potentials are aligned (*t* = 0) to the first spike in the burst. Black trace corresponds to a representative burst in the series illustrating precision of the isolated neuron’s fast dynamics.

The Komendantov-Kononenko model is a very rich dynamical model able to display regular, irregular or chaotic regimes as a function of the particular choice for the values of its parameters. Parameters in our simulations were set for a regular bursting regime (Table 1). With these values, the isolated neuron showed a highly stereotyped behavior, both in the slow and fast dynamics (Fig. 1). This allowed us to study whether some specific external stimulation led the neuron from a very regular and precise regime to new transient regimes defining a characteristic input-output transformation.

**Table 1:** Parameters of the Komendantov-Kononenko neuron model for the regular bursting regime used in our simulations.

*V*_*Na*_	*V*_*K*_	*V*_*B*_	*V*_*Ca*_	*C*_*m*_	*R*	*k*_*s*_	*ρ*	*k*_*β*_
40 mV	−70 mV	−58 mV	150 mV	0.02 *μ*F	0.1 mm	50 s^−1^	0.002	15000 mM^−1^
***β***	***g***_***k***_	***g***_***Na***_	***g***_***NaV***_	***g***_***B***_	***g***_***NaTTX***_	***g***_***KTEA***_	***g***_***Ca***_	***g***_***CaCa***_
0.00004 mM	0.25 *μ*S	0.02 *μ*S	0.105 *μ*S	0.105 *μ*S	400.0 *μ*S	10.0 *μ*S	1.5 *μ*S	0.02 *μ*S

#### Synaptic Model

To describe the synaptic input arriving at a neuron, we used the kinetic formalism for modeling chemical-mediated synaptic transmission proposed by Destexhe *et al*.^64,65^. Such framework has a demonstrated ability to capture the physiological properties of biological synapses mediated by different receptor types. In our simulations, we chose parameters to represent excitatory and inhibitory connections as AMPA- and GABA_A_-mediated synapses, respectively. Kinetics of both receptor types was simulated according to a two-state kinetic scheme^65^. Thus, synaptic input in the reader cell was represented as an additional current in the neuron model as:

18 $${I}_{syn}(t,V)=\sum _{i}\,{r}_{i}\,{g}_{sy{n}_{i}}\,(V-{E}_{syn})$$

where *i* represents the emitter neurons, ${g}_{sy{n}_{i}}$ is the maximal conductance of each connection; *V* is the postsynaptic potential (i.e., the membrane potential of the reader neuron); *E*_*syn*_ is the synaptic reversal potential; and the value of *r*_*i*_ gives the fraction of bound receptors and it is given by:

19 $$\frac{d{r}_{i}}{dt}={\alpha }_{syn}\mathrm{\ [}T]\,\mathrm{(1}-r)-{\beta }_{syn}\,r$$

being [T] the neurotransmitter concentration in the synaptic cleft; and *α*_*syn*_ and *β*_*syn*_ the forward and backward rate constants for transmitter binding, respectively. To compute the value of *r*, we assumed that [*T*] occurs as a spike-driven pulse initiated at the maximum voltage peak in the corresponding presynaptic unit and during which [*T*] = 1 mM. After that [*T*] = 0 mM.

### Stimulation

**Figure 2:**
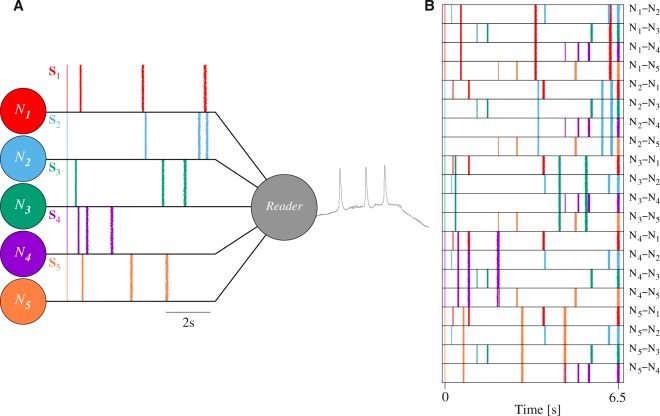
Schematic representation of the experimental setup. (**A**) *N*_1–5_ were bursting neurons acting cooperatively to produce sequential patterns of spiking-bursting activity. When they fired, they generated 4-spike bursts with a characteristic intraburst neural signature (*S*_*i*_). Raster plots characterizing signatures *S*_*i*_ contain 5000 bursts from the corresponding emitter. This graphical representation allows visually comparing the temporal structure of the five intraburst fingerprints (see also Table 2). The activity of neurons *N*_1–5_ was the input of the reader neuron, that computed an output in response to the presynaptic activation sequence. (**B**) 2-emitter activation sequences analyzed in our simulations (*N*_*i*_ − *N*_*j*_). Raster plots characterizing the spike timings in each sequence contain 3000 spike sequences aligned (*t* = 0) to the first spike in the sequence. The color code is the same as in panel A and identifies the spike source.

In a general scenario, neurons can receive thousands of inputs from multiple emitters. In our experiments, we considered the minimal setting illustrated in Fig. 2A oriented to investigate the arising of specific input-output relations from the simultaneous processing of signals from five emitters with a characteristic intraburst neural signature. The setup consisted of a reader cell connected to a group of emitter neurons (*N*_*i*_) that cooperatively produced coordinated sequential bursting activity. While the IBSP of all the bursts produced by a given emitter was always the same and different of the IBSP of the rest of emitters, i.e., the emitter cells had a characteristic intraburst signature (*S*_*i*_); the frequency, participants and phase relationships of the collective rhythm they generated at a given moment might change.

Using this simple experimental setup, we analyzed the reader’s response to different precise periodic inputs delivered in the context of the global sequential dynamics produced by neurons *N*_*i*_. The goal was to find preferred input-output relations in the form of stereotyped responses to specific activation sequences. Activation sequences differed in their spike timings and the input channel through which action potentials were delivered depending on the emitters that participated in the rhythm. For simplicity, the focus of our analysis was the input-output transformation during the processing of sequential patterns of activity in which the reader received coordinated input from two emitters, *N*_*i*_ and *N*_*j*_. The corresponding 2-emitter activation sequences were characterized by the combination of the emitters’ signatures, *S*_*i*_ + *S*_*j*_ (Fig. 2B). As the reader response could strongly depend on multiple features of the incoming spike sequences, to make our discussion more restrictive and isolate the effect of intraburst neural signatures from the information encoded in other aspects of the spiking-bursting input signals, we imposed every stimulation period to have the same duration and contained the same number of spikes.

## Results

As we were interested in the detection and distinct processing of combinations of intraburst neural signatures without any specific tuning in the synaptic connections, we analyzed and compared results of simulations performed with the same parameters for all the input channels of the reader neuron. In this paper, we focus on results corresponding to input patterns received through excitatory AMPA connections with ${g}_{sy{n}_{i}}=0.1\,\mu {\rm{S}}$, *E*_*syn*_ = 0 mV, *α*_*syn*_ = 0.5 ms^−1^ mM^−1^ and *β*_*syn*_ = 0.1 ms^−1^. Equivalent results to the ones presented here were obtained with GABA_A_-mediated inhibitory synapses (*E*_*syn*_ = −78 mV).

**Figure 3:**
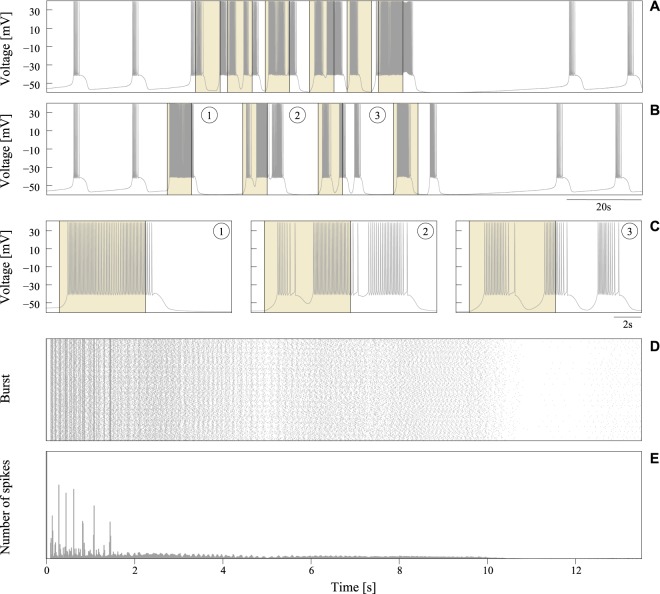
Response of the reader neuron to pairs of coordinated bursts with a random distribution of spikes. (**A**,**B**) Reader activity in response to a fast presynaptic rhythm (panel (A)) and a rhythm coherent with the slow-wave frequency of the reader (panel (B)). Shadowed areas identify the stimulation periods, defined as the time interval between the arrival of the first and the last spike in a spike input sequence (6.5 s in all cases, see main text for details). (**C**) Fragments of time series illustrating the different response of the reader neuron to the processing of two coordinated random bursts. Each trace corresponds to a stimulation cycle in panel (B). (**D**,**E**) Spike raster plot and PSTH characterizing the reader activity during 5000 consecutive random stimulation cycles. Spiking activity in these plots was aligned (*t* = 0) to the first postsynaptic spike fired after the stimulation.

As expected, when the reader cell was stimulated with a rhythmic input pattern of activity its behavior changed. Synaptic input elicited transient behavioral changes during the stimulation and a brief subsequent period (see panels A and B in Fig. 3). Taking into account that results presented in the following sections correspond to input received through excitatory synapses, note the increasing activity during the stimulation. When the stimulation was over, the neuron recovered back to the precise spiking-bursting behavior imposed by the cell intrinsic dynamics. These observations pointed to the correlation among the output spiking activity during the stimulation and the events in the input, since synaptic currents from the emitters were obviously the origin for the different behaviors displayed by the reader. In the following sections, we study whether specific combinations of neural signatures might elicit the generation of transient stereotyped responses underlying a characteristic input-output transformation in the cell.

### Stimulation without neural signatures

We first studied the response of the reader neuron to the reception of coordinated bursting signals that did not encode an intraburst neural signature. For that, we performed simulations where two presynaptic cells generated rhythmic patterns of bursting activity at different slow-wave frequencies. As they had not a characteristic fingerprint, their intraburst firing pattern varied randomly within each burst. The goal was to verify whether two neurons generating a precise bursting rhythm with a random IBSP – but equivalent regarding number of spikes and duration of the stimulation in each cycle – could lead to specific input-output relations in the reader.

Unlike the precise spiking-bursting activity generated by the isolated neuron (cf. Fig. 1), when this was stimulated with rhythmic bursting signals not encoding an intraburst signature, its response varied and became non-predictable. This occurred regardless the number of spikes in the input sequence, the time window between first and last spike, and the frequency of the presynaptic rhythm. Panels A and B in Fig. 3 illustrate this result displaying two representative examples of postsynaptic output when the reader processed sequences of 4-spike random bursts from two emitters producing a rhythmic pattern of activity at different frequencies. To generate these plots and allow comparison with results presented in the following sections, we took a special care to use a stimulation protocol equivalent to the one used below to discuss the processing of signals that encode a neural signature. In this way, we assured (i) that the reader received two 4-spike bursts through two different synaptic afferents within each rhythm cycle, and (ii) that the time interval between first and last spike in the input sequence was 6.5 seconds (see below). Given the bursting nature of the reader cell, a relevant feature pointing out the unpredictable input-output transformation during random stimulation was how postsynaptic spikes were grouped into bursts with different properties in each stimulation cycle. Panel C of Fig. 3 displays examples of time series showing the different organization of the output bursts in response to two 4-spike input bursts with random IBSPs, while panels D and E in this figure illustrate dispersion of the output raster plots and PSTHs during random stimulation.

The unpredictable response observed in the simulations with random bursts did not necessarily imply that the number of spikes and the duration of the stimulation in each cycle were not relevant informational aspects for the reader. The frequency of the presynaptic rhythm was an additional aspect to take into consideration in this regard, since the reader’ oscillation phase at the moment the input sequence was delivered could significantly vary depending on it (cf. panels A and B in Fig. 3). This variation in the phase might also be a plausible explanation for the unpredictable response in each stimulation cycle. To assess dependence of the output on the relationship between the sequence onset and the phase of the reader neuron, we performed new simulations where, departing from the same initial conditions, sequences of two bursts with random IBSPs and the same number of spikes and duration were delivered at a given fixed moment. We simulated the processing of multiple combinations of random sequences arriving at different phases of the reader oscillation. Regardless whether sequences arrived while the reader was firing or within the resting period between two consecutive bursts, the response was not predictable. Therefore, we concluded that neither the number of spikes in incoming bursts nor the time window between first and last spike in the sequence were key informational aspects for our reader cell to compute a selective response.

### Detection of activation sequences by means of neural signatures

The next step in our investigation was to study the emergence of preferred neuronal input-output relations in response to sequential bursting activity encoding an intraburst fingerprint. We focused on the processing of sequences containing the same number of spikes and with the same duration. In this way, we isolated the effect of neural signatures in the input-output transformation from the effect of these additional informational aspects in the bursting activity.

The first result derived from simulations where the emitters had a characteristic neural signature was a strong dependence of the reader’s output on the slow-wave frequency of the presynaptic rhythm. In our analysis, we distinguished three situations as a function of the relationship between this frequency and the reader slow dynamics. With fast rhythms (as compared with the reader’s activity), input sequences usually arrived while the reader was firing a burst because of the processing of a previous input. In this situation, the same as in the case of input signals with random IBSPs (Fig. 3A), the reader produced unpredictable responses. This result highlighted again relevance to compute the response of the reader phase at the moment the sequence was delivered. For slow rhythms, the situation was similar in the sense that some sequences arrived while the reader was firing, in this case because of its intrinsic dynamics. Responses to these sequences were not predictable either. In terms of this work, these results implied that the reader did not implement any preferred input-output relation for presynaptic rhythms non-coherent with its slow dynamics (i.e., much faster or slower rhythms). However, the most relevant situation from the perspective of our investigation was the processing of rhythms coherent with the reader’s activity. First, because this is the most typical situation in the context of the circuits where intraburst neural signatures are present (e.g., CPG circuits). Second, because these rhythms minimized the effect of the interplay between the reader phase and the sequence onset on the postsynaptic response. As the frequency of the presynaptic rhythm became closer to the reader’s oscillation frequency, as opposed to what happened with faster rhythms, the reader was able to reach the resting period before the arrival of new inputs (e.g., see Fig. 3B). And, as opposed to what happened with slower rhythms, sequences in the series – maybe except the first one – were always delivered within the resting period between bursts. In this situation, some specific combinations of intraburst neural signatures elicited a highly stereotyped and characteristic output in the reader. In this regard, it is important to highlight that not all the intraburst ISI distributions allowed a selective input-output transformation.

As representative example of the emergence of preferred input-output relations in response to sequential bursting activity encoding an intraburst fingerprint, we analyze and discuss here simulations with five emitter neurons (*N*_1_–*N*_5_) producing bursts with four spikes (half than the bursts produced by the isolated neuron, Fig. 1B). The signature (*S*_*i*_) of each emitter (*N*_*i*_) was given by the following intraburst ISI distribution (units are seconds):

- *S*_1_ = {*ISI*_1_ = 0.60 ± 0.02, *ISI*_2_ = 2.80 ± 0.02, *ISI*_3_ = 2.80 ± 0.02}
- *S*_2_ = {*ISI*_1_ = 3.50 ± 0.02, *ISI*_2_ = 2.40 ± 0.02, *ISI*_3_ = 0.35 ± 0.02}
- *S*_3_ = {*ISI*_1_ = 0.40 ± 0.02, *ISI*_2_ = 3.90 ± 0.02, *ISI*_3_ = 1.00 ± 0.02}
- *S*_4_ = {*ISI*_1_ = 0.50 ± 0.02, *ISI*_2_ = 0.40 ± 0.02, *ISI*_3_ = 1.10 ± 0.02}
- *S*_5_ = {*ISI*_1_ = 0.70 ± 0.02, *ISI*_2_ = 2.20 ± 0.02, *ISI*_3_ = 1.60 ± 0.02}

Note that a small dispersion was introduced in the ISI distributions to produce a realistic temporal variation in the intraburst spiking activity^18,66–68^. Figure 2A represents these five signatures as raster plots aligned to the first spike in the burst, and Table 2 quantifies the distance ${d}_{{S}_{i},{S}_{j}}$ (Eq. 1) between signals from each possible pair of emitter cells.

**Table 2:** Distance between the intraburst neural signature of the five emitter cells of Fig. 2A calculated with Eq. 1.

	*S*_1_	*S*_2_	*S*_3_	*S*_4_	*S*_5_
***S***_**1**_	8.02 · 10^−4^	—	—	—	—
***S***_**2**_	14.6	8.04 · 10^−4^	—	—	—
***S***_**3**_	4.49	12.3	8.05 · 10^−4^	—	—
***S***_**4**_	8.66	13.6	12.3	7.95 · 10^−4^	—
***S***_**5**_	1.81	9.44	3.34	3.53	8.04 · 10^−4^

These metrics were calculated comparing time series with 5000 bursts each. The distance of each signature to itself is shown to give a reference value for the similitude measurement.

**Figure 4:**
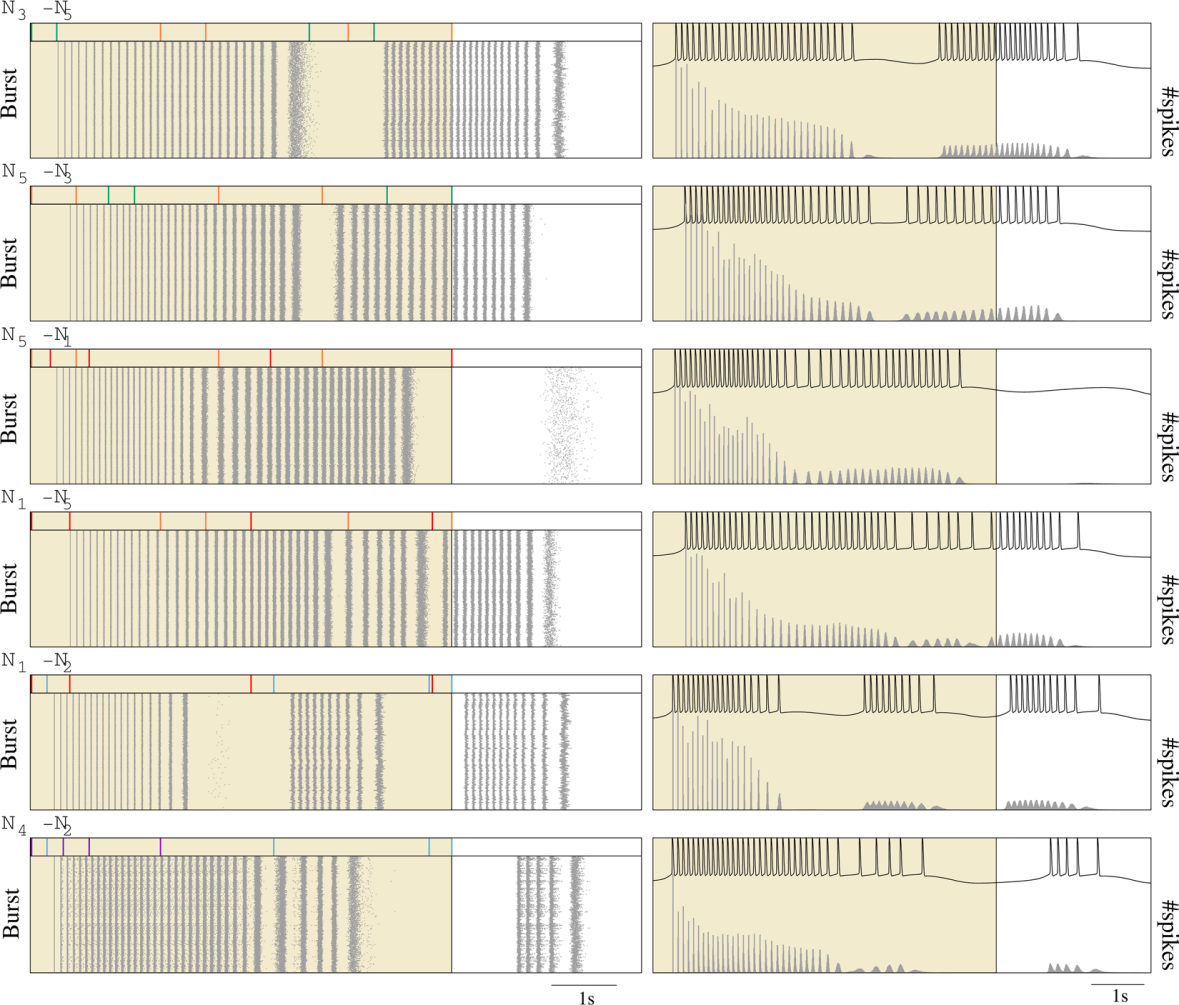
Stereotyped response of the reader to different 2-emitter activation sequences from *N*_1–5_. Spike raster plots (left) and PSTHs (right) were generated considering 5000 consecutive stimulation periods – defined as the time interval between the arrival of the first and the last spike in an input sequence (shadowed areas). Postsynaptic activity was aligned to the first spike produced during the stimulation. In contrast to previous figures, the delay from delivery of the first spike in the input to the generation of the first spike in the output is now included both in the raster plots and the PSTHs. Panels on top of the raster plots display the input spike sequence in each rhythm. The color code used to identify the spike source is the same used in Fig. 2A. Note the precise output as compared to the random stimulation (cf. Fig. 3), and the characteristic stereotyped response for each pair of signals. Time series above PSTHs show an example of these characteristic responses.

**Figure 5:**
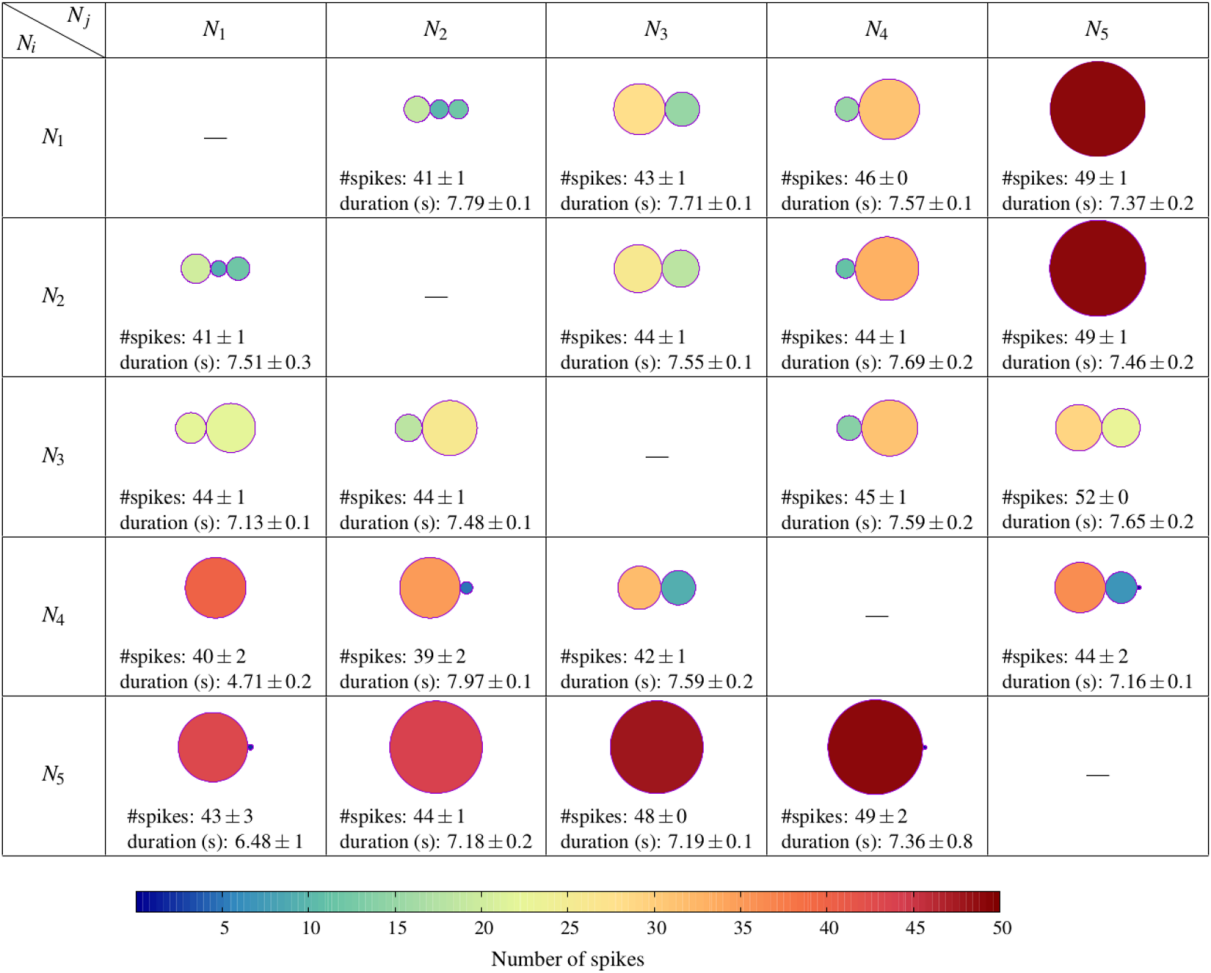
Characterization of the reader output as a function of the emitters participating in the input rhythm, i.e., of the combination of signatures processed by the neuron in a stimulation cycle. Circles represent series of bursts produced in the output in response to a given input sequence (*N*_*i*_ − *N*_*j*_): from 1 to 3 response bursts depending on the input. The size and the color of the circles (see color-map at the bottom) represent the mean duration and the mean number of spikes in the corresponding output burst, respectively. Note that this representation does not allow the comparison of the intraburst temporal structure of the bursts. For this, we use the output raster plots and PSTHs. Data included in each cell correspond to the mean number of bursts produced in response to the corresponding activation sequence, the mean total number of spikes and the mean total duration of the response – computed as the time interval between the first and the last output spike produced in response to the input sequence.

When the reader processed rhythmic patterns of bursting activity generated by two of the neurons *N*_1–5_, it synchronized with the emitters. This was in line with the classical view on bursting activity, pointing out that the slow depolarizing bursting period was relevant to compute the reader’s output. However, these rhythms also led to preferred input-output relations as a function of the source of the signals building up the coordinated pattern activity. This selective input-output transformation was independent of the presynaptic slow-wave frequency and the reader phase when the sequence arrived. Figure 4 shows examples of the selective response to distinct biphasic rhythms produced by neurons *N*_1–5_. In particular, raster plots (left panels) and PSTHs (right panels) in this figure characterize the corresponding output spike timings. Note that the sequences processed in simulations of Fig. 4 were equivalent regarding number of spikes (*n* = 8), duration of the stimulation (6.5 s) and slow-wave frequency to those shown in Fig. 3B–E. The only difference was the encoding of an intraburst signature which underlay the precise presynaptic IBSPs. The temporal structure of the spikes produced in the output significantly varied depending on the emitters participating in the input rhythm: from sequences of nearly regular bursts to sequences of accelerating or decelerating bursts. There were also differences regarding number of spikes, duration, phase and/or number of response bursts. Figure 5 quantifies these differences for all the possible biphasic rhythms produced by neurons *N*_1–5_.

Since all the properties of the presynaptic rhythms but the signatures identifying the origin of the input signals were the same, we might assume that the emerging preferred input-output relations appeared due to the presence of these precise temporal structures. However, the selective respond of the reader did not depend on the encoding of a given signature in the input, i.e., there was not a correlation between the generation of a given stereotyped output and the activation of a specific emitter. For instance, in four of the examples shown in Fig. 4 (see also Fig. 5), the reader received input from *N*_5_ and, therefore, processed signature *S*_5_. In all these cases, the neuron response varied – from the generation of two bursts to the generation of a single longer or a single shorter burst – as a function of the signature encoded in the additional signal received during the stimulation cycle. This pointed to the combination of incoming signatures as responsible for the selective input-output transformation. Additionally, the order of presentation of these signatures was also relevant for determining the stereotyped response produced by the reader. Response to *S*_*i*_ + *S*_*j*_ was different to response to *S*_*j*_ + *S*_*i*_ (cf. responses to *N*_3_ − *N*_5_ and *N*_5_ − *N*_3_, or to *N*_5_ − *N*_1_ and *N*_1_ − *N*_5_ in Fig. 4). This was highly relevant for the characteristic input-output transformation discussed in this paper, since the relative arrival timing of the bursts encoding the neural signatures determined the precise activation sequence among the emitters (*N*_*i*_ followed to *N*_*j*_ or vice versa).

**Figure 6:**
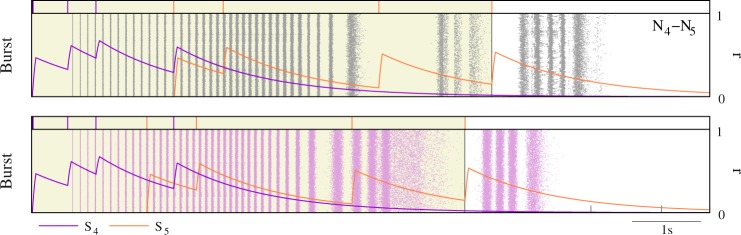
Response of the reader to a temporal shift in the activation sequence. Raster plots equivalent to the ones shown in Fig. 4 illustrating the different response of the reader to the activation sequence *N*_4_ − *N*_5_ (top) when the activity of *N*_5_ was slightly anticipated in relative to bursts from *N*_4_ (bottom). Purple and orange traces show temporal evolution of the variable *r* corresponding to the connection between the reader and neurons *N*_4_ and *N*_5_, respectively.

**Figure 7:**
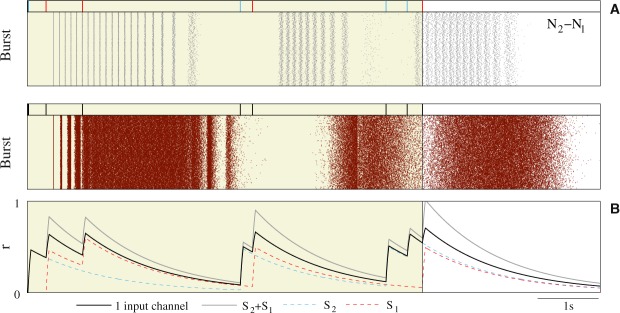
Comparison of the reader response to two intraburst signatures and to a single burst with an equivalent IBSP. (**A**) Output raster plots equivalent to the ones shown in previous figures, in this case characterizing the stereotyped response produced when the reader processed the activation sequence *N*_2_ − *N*_1_ (top) and the non-predictable response to sequences of 8-spike bursts from a single presynaptic unit with an IBSP equivalent to *S*_2_ + *S*_1_ (bottom). (**B**) Trajectories of the corresponding synaptic variables *r* in response to a representative input spike pattern in each case. Gray trace corresponds to the combined action of signatures *S*_2_ and *S*_1_ in the synaptic cleft. Action potentials from *N*_1_ (cyan) and *N*_2_ (red) were delivered through a different synaptic channel (dotted traces). Black trace corresponds to the single emitter case.

Results confirming the selective input-output transformation as a function of the identification of activation sequences by means of the recognition of intraburst signatures were observed in simulations (i) where the bursting activity of the second active emitter was slightly anticipated or delayed (Fig. 6), or (ii) where a combination of presynaptic signals defined an input spike pattern equivalent to *S*_*i*_ + *S*_*j*_ but with some spike delivered through a different synapse (e.g., eight single spikes delivered through eight different input channels or 8-spike bursts arriving through a single channel, Fig. 7). If we compared the reader’s response in these simulations with the corresponding stereotyped response produced when processing *S*_*i*_ + *S*_*j*_, the output changed and even became unpredictable. These simulations also served to illustrate the underlying mechanisms behind the recognition of specific combinations of intraburst signatures. With this aim, Figs 6 and 7 display the trajectories of the synaptic variables within a stimulation cycle. The analysis of these trajectories revealed relevance to compute the output of the interplay among the multiple time scales involved in the synaptic processes, the intrinsic dynamics of the reader neuron and the temporal structure of the inputs. These complex interactions could give rise to highly different input-output transformations with only a small variation in the input spike timings (e.g., compare top and bottom panel in Fig. 6). Even in the case of action potentials arriving at the same relative timing but delivered through different afferents, the interplay among these temporal dynamics played a critical role in the computation of the output. In particular, synaptic temporal dynamics induced significant changes in the evolution of the total fraction of bound receptors during the stimulation depending on the stimulation history of the synaptic afferent. This underlay the non-linear sum of synaptic currents responsible of producing different responses in each case (cf. gray and black traces in Fig. 7). These results reflected the complex interaction among the resonant processes involved in the computation of the model neuron response, suggesting that intraburst neural signatures, whose constituent spikes must be delivered through the same input channel, could not only allow a postsynaptic neuron to react selectively to specific activation sequences among its presynaptic partners, but also to precise phase relationships among the active cells.

**Figure 8:**
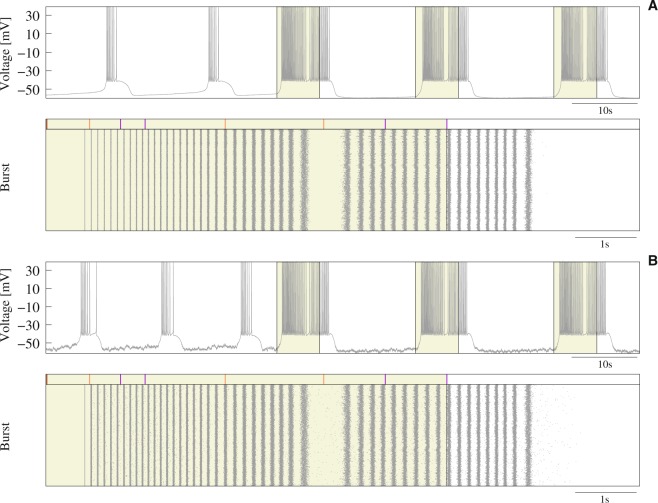
Robustness to noise of the discussed phenomena. (**A**) Response of the reader to the processing of the activation sequence *N*_5_ − *N*_4_ in a simulation without noise. (**B**) Response to the same activation sequence in a simulation with a maximum noise amplitude equal to 0.44 (around 20% of the maximal synaptic current received by the reader). Note that although noise affected the frequency and IBSP of the reader in the absence of stimulation (first part of time series), it produced the same stereotyped response to *S*_5_ + *S*_4_.

Finally, to assess robustness of the detection of activation sequences, we introduced in Eq. 2 additive white noise computed from a uniform distribution on the interval [0, *max*_*noise*_] at each time step, and performed simulations with different levels of noise as indicated by the value of *max*_*noise*_. All discussed phenomena occurred both in the presence and absence of noise, even for levels of noise affecting the precise slow and fast dynamics of the isolated neuron (Fig. 8) or leading it to a chaotic spiking regime. Simulations with a maximum noise level below 30% of the maximal synaptic current received by the reader produced the same results of the simulations presented in this paper. For higher levels (up to 43% of the maximum synaptic current), stereotyped responses to each combination of signatures could change, but results were equivalent. Results of these simulations pointed out the robustness of the discussed phenomena.

## Discussion

Neural signatures are robust and cell-specific intraburst firing patterns that can be found in different bursting neurons^18–22^. Although the existence of these fingerprints does not guarantee that nervous systems make use of such temporally precise code, information processing based on their identification can be a powerful strategy for neural systems to enhance their capacity and performance. In this paper, we have studied these neuron-specific temporal structures from a functional point of view. We show that a neuron conductance-based model is able to take advantage of them to identify the origin of its input signals and sensitively adjust its response to specific activation sequences. The response of our reader cell varies as a function of different features of the input, in particular, as a function of the frequency of the presynaptic rhythm, the number of spikes in incoming bursts and the duration of the stimulation. In our study, we take a special care to isolate the effect of intraburst neural signatures from these additional informational aspects of the spiking-bursting signals. The reader response has anyway a strong dependence on the slow-wave frequency of the presynaptic activity. Depending on this frequency, the oscillation phase of the reader at the moment the input sequence arrives is one of the main factors determining the output. Nevertheless, for presynaptic rhythms coherent with the reader activity, if the signals that constitute the input sequential pattern encode a characteristic signature identifying their source, the output does not depend on the reader’s oscillation phase. In this situation, specific combinations of intraburst fingerprints lead to complex preferred input-output relations, and the reader produces characteristic stereotyped bursting responses depending on the cells participating in the rhythm and the precise phase relationship among their bursting activity. Changes in the firing order of the emitters, in their IBSPs, in their timings within the sequence or in the synaptic afferent through which action potentials are delivered induce changes in the output. The emergence of such preferred input-output relations supports the hypothesis of the recognition of specific activation sequences by means of the emitters’ characteristic intraburst signature. Besides this, the reader synchronizes in any case with the presynaptic rhythm, which highlights that it uses the information encoded in different aspects of the input signals to compute its output.

Relevance of detecting activation sequences becomes apparent when we consider neurons at the population level. When a group of neurons working together to perform a given task produces precise and reliable spike trains, the neurons they are connected with receive sequential trains of spikes from different sources^69–72^. Robust sequences of neural activations have been described in many invertebrate and vertebrate systems^42–58^. These sequences play a critical role to encode, control and execute information in different sensory, central and motor networks^73–77^. In this scenario, the preferred input-output relations discussed in this paper would allow some specialized readers to build context dependent responses as a function of certain relevant activation sequences, while other readers keep blind to intraburst signatures and completely ignore these temporal structures in their input signals. This selective input-output transformation can result in information discrimination mechanisms associated to the generation and coordination of sequential dynamics. For example, even the rhythms generated by simple CPG circuits are highly flexible^78,79^. On one hand, the shape and phase relationships of the electrical activity of CPG neurons are continuously adapted to an ever changing environment^41,80–84^. On the other hand, many CPGs are multifunctional networks capable of switching between more than one behavior depending on the particular circumstances under which the circuit is working^76,85–88^. Behavioral changes of a CPG are related, for instance, to variations of the slow depolarizing frequency, of the firing timings among the interacting elements or of the participants in the sequential pattern of activity. The properties of the rhythm generated by the network at a given moment arise from the combination of the intrinsic properties of each individual cell, the connection topology of the network, the properties of the synaptic connections and the modulatory inputs^44,89–93^. Our results suggest that the ability of single CPG neurons to discriminate different activation sequences could be an additional factor contributing to shape the resultant pattern during the rhythm negotiation. Additionally, external readers of an ongoing CPG rhythm could identify changes in the activation sequence of the interacting neurons, and consequently adapt their behavior to these changes.

A relevant question regarding the emergence of the preferred input-output relations discussed in this paper is what mechanisms could underlie the detection and discrimination of different activation sequences by means of the emitters’ intraburst neural signature. There is not a simple answer to this question. The recognition of intraburst fingerprints requires of history-dependent processing capabilities in the postsynaptic cell. History-dependent processing capabilities offered by intrinsic neural dynamics have long been investigated and, currently, it is well-known that neurons can carry information about its history of stimulation through its dynamical variables. In particular, certain ionic currents have been considered as molecular basis for single-cell transient memory due to the history dependence on the dynamics of the corresponding ionic channels^94–97^. Previous computational studies with Hodgkin-Huxley type models^23^ suggest that this could be the single-neuron substrate of history-dependent processing for the recognition of neural signatures in CPG cells. In particular, kinetics of the calcium-dependent channels affects the response to a signal encoding a particular signature. However, our work here hints at the synaptic dynamics as an additional factor to take into consideration. Our results suggest that the recognition of different sequences depends on the complex interaction among the multiple time scales involved in the processing of incoming signals through the different input channels and the reader intrinsic dynamics, which reshapes the resonant properties of the neuron. However, additional biophysical mechanisms proposed in literature as candidates for decoding precise temporal codes at the single-cell level (e.g, see refs^14–16,98–102^) could also apply for the recognition of neural signatures and activation sequences in living cells.

Finally, we would like to highlight that, although we have mainly related our work to CPG sequential dynamics, our results are not limited to these networks. As we have previously pointed out, sequential dynamics can be found in almost any vertebrate system linked to complex behaviors. Even most brain rhythms, typically characterized by their frequency, are built from sequential activations of different groups of neurons^103,104^. Precise timings in the spiking activity of multiple neurons have also been reported in widely different invertebrate and vertebrate neural systems (e.g., see refs^105–111^). Furthermore, CPGs are valuable biological models for investigating and understanding neural dynamics^112^. Findings on these simple neural networks have proven to be generalized to more complex networks in order to explain the computational properties of the nervous system^41,45,113^. In this line, they have been proposed as a conceptual framework for understanding cortical microcircuits (i.e., functional ensembles of neurons) because of their morphological and dynamical properties^114^. In this way, coordination mechanisms based on the emission and recognition of neural signatures can arise, for instance, in the vertebrates’ spinal cord, where multiple bursting neurons work together to generate rhythmic patterns of activity in a hierarchical motor network^72,115–118^. Therefore, we speculate that the detection of activation sequences by means of the recognition of characteristic neural signatures can have a place in the arsenal of strategies of information processing in the nervous system.
